# Standardized Criteria to Initiate External Ventricular Drain (EVD) Weaning in a Neurological Intensive Care Unit to Increase the Safety of EVD Discontinuation and Reduce the Need for a Shunt

**DOI:** 10.7759/cureus.58362

**Published:** 2024-04-16

**Authors:** Sachin A Kothari, Mevish S Siddiq, Scott Rahimi, Fernando Vale, Manan Shah, Klepper Alfredo Garcia

**Affiliations:** 1 Neurology, University of Chicago Medical Center, Chicago, USA; 2 Neurology, Columbia University Irving Medical Center, New York, USA; 3 Neurosurgery, Augusta University Medical College of Georgia, Augusta, USA; 4 Neurology, Augusta University Medical College of Georgia, Augusta, USA

**Keywords:** neuro-critical care, endoscopic third ventriculostomy, ventriculoperitoneal shunt, external ventricular drain, subarachnoid hemorrhage

## Abstract

Introduction

Patients with subarachnoid hemorrhages (SAH) with external ventricular drains (EVD) can develop chronic hydrocephalus (HCP), requiring permanent cerebrospinal fluid (CSF) diversion via an external shunt. Two different strategies have been used to assess for dependence on EVD: 1) prompt closure, and 2) gradual weaning. Gradual weaning of EVDs is performed by increasing drainage resistance to outflow over days. However, when to start one strategy or the other is up to the physician. No uniform guidelines exist raising a question: Are standardized criteria necessary to initiate the EVD weaning process for SAH patients to increase the safety of EVD discontinuation and reduce the need for a shunt?

This study shares criteria used to initiate EVD weaning that displayed increased safety of EVD discontinuation for patients with subarachnoid hemorrhage requiring EVD, particularly with regards to length of hospital stay (LOS), hospital-acquired infection rates, and ventriculoperitoneal shunt/endoscopic third ventriculostomy (VPS/ETV) placement.

Methods

One hundred and fifty-one SAH patients from January 2016 to January 2019 were analyzed. 60 aneurysmal SAH (aSAH) and 18 non-aneurysmal nontraumatic SAH (naSAH) patients required EVD placement. A gradual EVD weaning protocol was initiated if patients met the following criteria: 1. The reason for EVD placement has resolved or is resolving, 2. The quantity of CSF output is <250mL over 24 hours, 3. Quality of CSF is nonbloody, 4. Intracranial Pressure (ICP) must be within normal limits, and 5. The patient must be neurologically stable. It was acceptable to initiate the weaning process when the patient had mild cerebral vasospasm, but not moderate to severe cerebral vasospasm. EVD weaning was performed by increasing the drain (chamber) height by 5 millimeters of mercury every 24 hours if the criteria were met. Charts were reviewed for LOS, infection rates, and rate of VPS/ETV. Gender, age, race, wean failure incidence, Hunt-Hess scores, modified Fisher scores, and syndrome of inappropriate antidiuretic hormone/cerebral salt wasting (SIADH/CSW) rates were obtained.

Results

The average LOS for aSAH patients with EVD was 20.35 days. The incidence of VPS/ETV was 11%. A chi-square analysis revealed that aSAH patients had higher rates of VPS/ETV placement (p<0.001) and EVD wean failures (p<0.001) than naSAH patients. aSAH patients had a lower incidence of VPS/ETV placement of 11% compared to 21% nationally.

Conclusions

Standardized criteria to initiate EVD weaning provided a reduction in VPS/ETV placement among aSAH patients compared to national averages and provided a uniform approach to EVD management. Comparable infection rates and LOS for SAH patients requiring EVDs compared to national averages were found.

## Introduction

A subarachnoid hemorrhage (SAH) is a type of stroke in which there is bleeding into the subarachnoid space. It can be further classified as traumatic or non-traumatic (spontaneous) SAH. Non-traumatic SAH can be further divided into aneurysmal or non-aneurysmal [1]. Acute hydrocephalus requiring temporary cerebrospinal fluid diversion occurs in between 15% to 87% of subarachnoid hemorrhage patients. External ventricular drains (EVD) placement for cerebrospinal fluid (CSF) diversion is often used to treat hydrocephalus and increased intracranial pressure often seen in SAH patients [2-4]. However, when it is time to wean the EVD with the goal of EVD removal, the weaning process can fail, leading to the placement of a ventriculoperitoneal shunt (VPS) or endoscopic third ventriculostomy (ETV) [5]. The Neurocritical Care Society in a consensus statement urged that EVD weaning should be done “as quickly as is clinically feasible” [6]. However, standardized guidelines to begin EVD weaning are limited [7]. This is a critical issue and can have numerous implications which this study aims to analyze. Lack of standardization often leads to delays in EVD weaning which affects the length of stay in the ICU and hospital. This, in turn, is associated with increased cost/resources and increased hospital-acquired infection rates in subarachnoid hemorrhage patients [8-10].

The purpose of this paper is to share a simplified EVD weaning initiation criteria developed over the course of 8 years that has reduced EVD wean failures and subsequently reduced rates of VPS/ETV placement at our institution compared to national averages.

## Materials and methods

Study population

This study is a single-center observational study with retrospective data collection. The participating site is a designated comprehensive stroke center that maintains data of all subarachnoid patients per requirements set forth by the American Heart Association. We screened the database for patients with nontraumatic subarachnoid hemorrhage patients admitted between January 2016 and January 2019. 151 patients were identified of which 78 required EVD placement (60 of these patients had aneurysmal SAH or aSAH and 18 had a nonaneurysmal subarachnoid hemorrhage or naSAH). Patient charts were reviewed, and the following parameters were analyzed: age, gender, race, ICU length of stay (LOS), infection rates, intracranial pressure (ICP), CSF output, number of days on EVD, EVD weaning failure incidence, syndrome of inappropriate antidiuretic hormone (SIADH)/cerebral salt wasting (CSW), and VPS/ETV placement.

Statistical methods

Univariate analysis was performed using chi-square analysis for categorical data and independent samples t-test for continuous data. To compare inter-institutional VPS shunt rates, a one-sided z-test was performed. Statistical analysis was performed using Microsoft Excel (Microsoft Corporation, Redmond, USA). A value of p < 0.05 was considered significant.

Criteria to initiate EVD weaning

A gradual EVD weaning protocol was implemented if patients met the following criteria: (1) The reason for EVD placement has resolved or is resolving, (2) the quantity of CSF output is <250mL over 24 hours, (3) the quality of CSF is non-bloody, (4) ICP is within normal limits, and (5) the patient is neurologically stable. It was acceptable to wean when the patient had mild cerebral vasospasm, but not moderate to severe cerebral vasospasm. EVD weaning was performed by increasing the drain (chamber) height by 5 millimeters of mercury (mmHg) every 24 hours if criteria were met, until it reached 20 mmHg after which the drain was clamped, although this could be performed every 12 hours instead of 24 hours [10]. After clamping for 24 hours, a head CT was obtained and the EVD was discontinued if there was no evidence of hydrocephalus. In our analysis, an EVD wean failure was defined as having at least one incidence in which the EVD drain height needed to be lowered during the weaning process.

## Results

Of 151 SAH patients, 100 patients had an aSAH while 51 patients had a naSAH. 78 patients required EVD placement and were included in the final analysis (60 in the aSAH group and 18 in the naSAH group). The mean age of the aSAH group was 59.62 years with 75.0% females; the mean age of the naSAH group was 54.94 years with 33.3% females. Most patients in both groups were Caucasian (51.7% in aSAH, 61.1% in naSAH). Average Hunt and Hess scores were 3.38 for aSAH and 2.94 for naSAH. Average modified Fisher scores were 3.75 for aSAH and 3.72 for naSAH. The average length of stay for aSAH patients was 15.5 days (Range: 0-49 days) and 20.35 days for ICU (Range: 0-48 days) and total hospital LOS, respectively. The average length of stay for naSAH patients was 16.56 days (Range: 8-36 days) and 20.00 days (Range: 6-32 days) for ICU and total hospital LOS, respectively. The average EVD placement length was 14.02 days for aSAH and 15.06 for naSAH. The average total EVD output was 2197.28 mL and 2791.0 mL for aSAH and naSAH, respectively. The average daily EVD output was not measured. 18 aSAH patients and 7 naSAH developed SIADH/CSW. Average daily ICP was found to be 10.06 mmHg (Range: -2.88 - 31.12) (aSAH patients) and 10.20 mmHg (Range: 6.59 - 16.38) (naSAH patients). The average daily change in ICP was found to be 13.20 mmHg and 12.88 mmHg for SAH and naSAH. These variables have been summarized in Table 1. 

**Table 1:** Demographics of aneurysmal versus non-aneurysmal subarachnoid hemorrhagic patients Table 1 displays the demographics of aneurysmal (aSAH) versus non-aneurysmal subarachnoid hemorrhage (naSAH) patients. There were 60 aSAH patients and 18 naSAH patients.  Data has been represented as Mean ± Standard Deviation. LOS: length of stay; EVD:  external ventricular drain; SIADH/CSW: syndrome of inappropriate antidiuretic hormone/cerebral salt wasting; ICP: intracranial pressure

	Aneurysmal SAH (N = 60)	Non-Aneurysmal SAH (N = 18)
Female	45	6
Mean Age ± SD	59.62 ± 12.78	54.94 ± 14.09
Race: Caucasian, African American, Asian	31, 27, 2	11, 7, 0
Mean Hunt-Hess Score ± SD	3.38 ± 1.06	2.94 ± 0.80
Mean Modified Fisher Score ± SD	3.75 ± 0.57	3.72 ± 0.57
Mean ICU LOS (days) ± SD	15.50 ± 9.23	16.56 ± 7.41
Mean Hospital LOS ± SD	20.35 ± 12.00	20.00 ± 8.21
Mean Length on EVD (days) ± SD	14.02 ± 7.61	15.06 ± 6.20
Patients with SIADH/CSW	18	7
Mean Daily Intracranial Pressure (mmHg) ± SD	10.06 ± 4.44	10.20 ± 2.26
Mean Daily change in ICP (mmHg) ± SD	13.20 ± 6.19	12.88 ± 2.59
Mean Total EVD Output (mL) ± SD	2197.28 ± 1503.75	2791.00 ± 1781.60

The incidence of VPS/ETV was 11%. Chi-square analysis was performed, and aSAH patients were found to have higher rates of VPS/ETV placement (p<0.001) and EVD wean failures (p<0.001) than naSAH patients. The characteristics of the study population at our institution are summarized in Table 1, which helps highlight that aSAH patients had higher rates of both VPS/ETV and EVD wean failures. 

**Figure 1:**
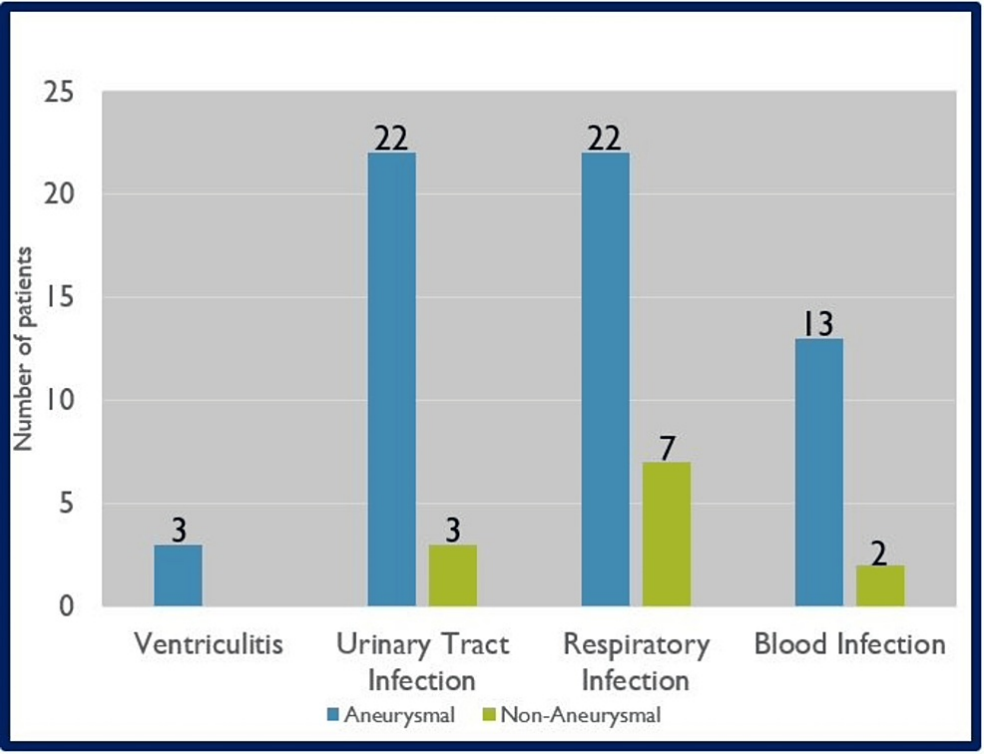
Incidence of infection in SAH patients with EVDs This figure provides a breakdown of the incidence of infection in subarachnoid hemorrhage (SAH) patients with an external ventricular drain (EVD).

**Figure 2:**
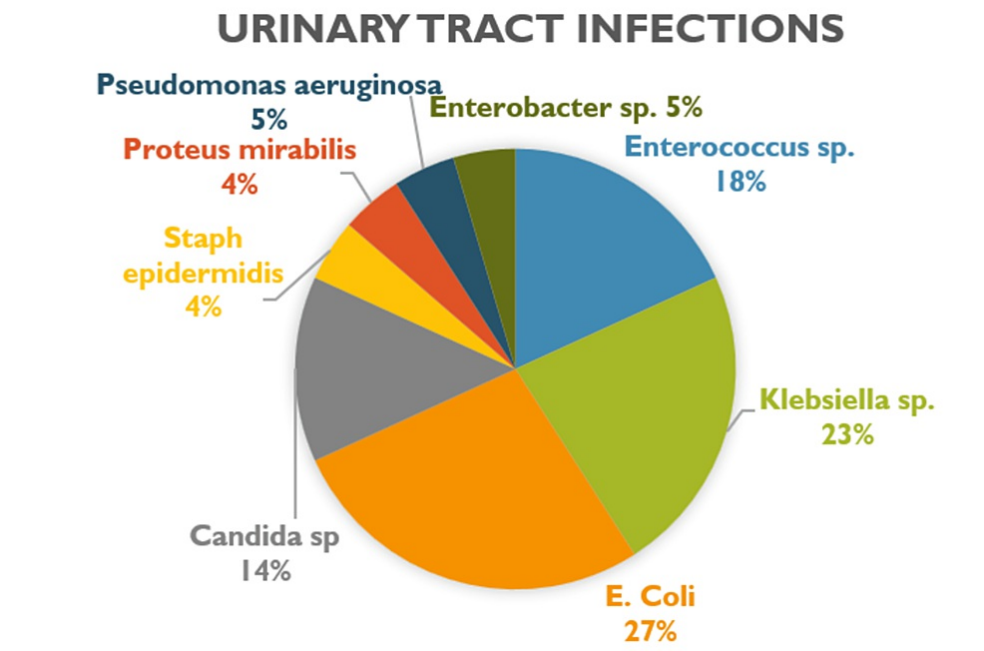
Microbes found in urinary tract infections in SAH patients with EVD. This figure provides a breakdown of the different microbes that were discovered in patients with subarachnoid hemorrhage (SAH) and external ventricular drain (EVD) with urinary tract infections. E. Coli: *Escherichia coli*; Stap epidermis: *Staphylococcus epidermidis*

**Figure 3:**
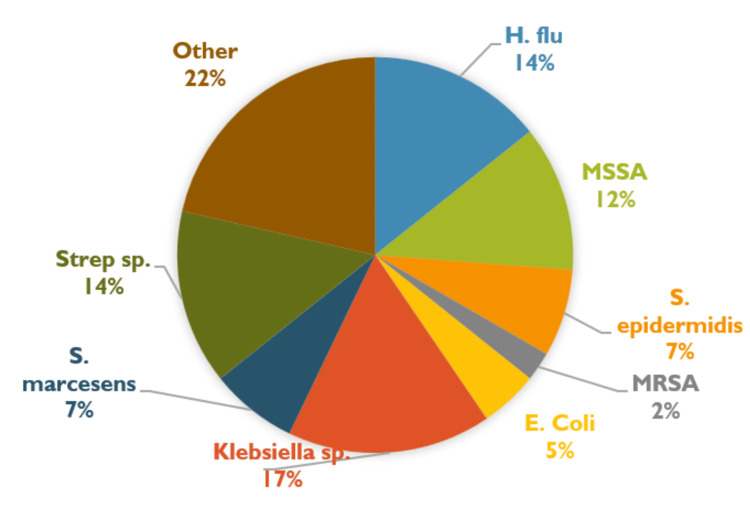
Microbes found in lower respiratory tract infections in SAH patients with EVD This figure provides a breakdown of the different microbes that were discovered in patients with subarachnoid hemorrhage (SAH) and external ventricular drain (EVD) with lower respiratory tract infections. MRSA: methicillin-resistant *Staphylococcus aureus*; MSSA: methicillin-sensitive *Staphylococcus aureus*; S. epidermis: *Staphylococcus epidermidis*; H. flu: *Haemophilus influenzae*; Strep sp.: *Streptococcus sp.*; S. marcescens: *Serratia marcescens*; E. Coli: *Escherichia coli*

The incidence of infections in EVD patients was also measured. Among aSAH patients, three developed ventriculitis, 22 developed urinary tract infections (UTIs; 20 females, two males), and 22 developed lower respiratory infections. Among naSAH patients, three developed UTIs (two females, one male), and seven developed lower respiratory infections. *Escherichia coli *was found to be the predominant bacteria in UTIs (27%) followed by *Klebsiella sp.* (22%). *Klebsiella sp. *(17%) was found to be the predominant bacteria for lower respiratory infections followed by *Streptococcus sp.* (14%). These findings have been represented in Figures 1, 2, 3 displaying that aSAH patients with EVD had higher rates of infections.

**Figure 4:**
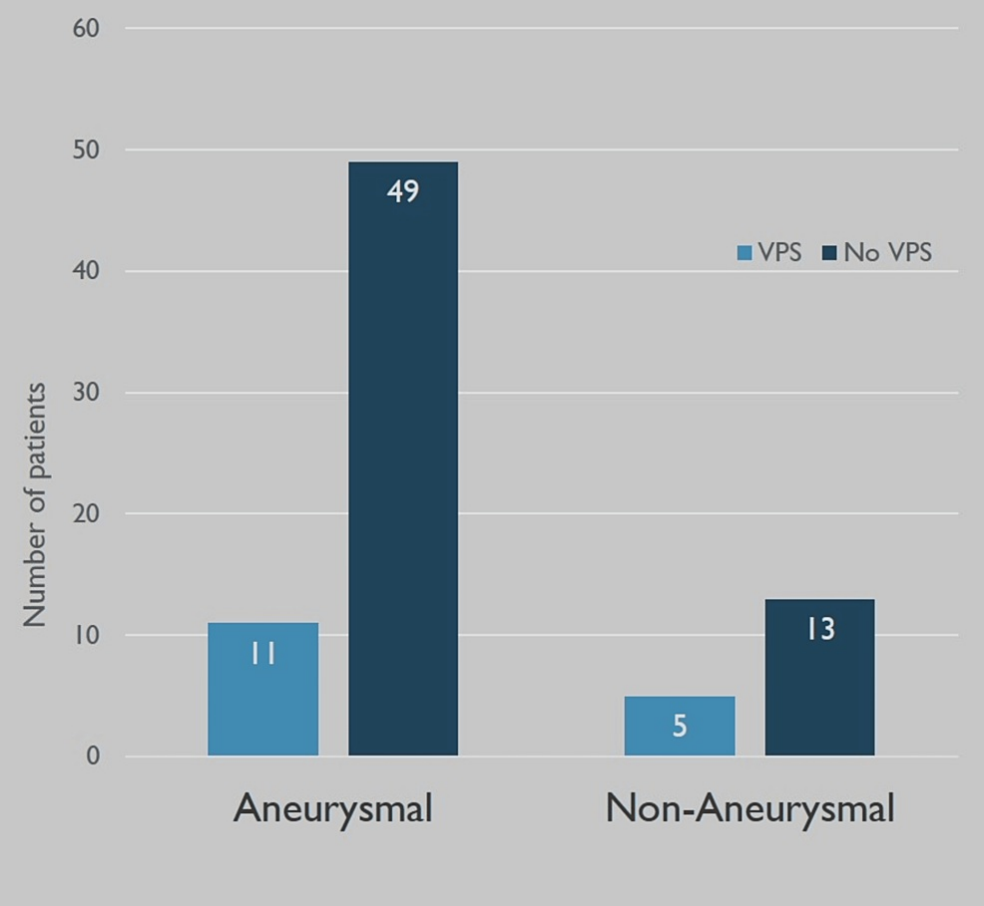
Rates of ventriculoperitoneal shunt placement in aSAH and naSAH with EVD patients This figure presents ventriculoperitoneal shunt placement in both aneurysmal subarachnoid hemorrhage (aSAH) and non-aneurysmal subarachnoid hemorrhage (nsSAH) patients with external ventricular drains (EVD) (p < 0.001).

**Figure 5:**
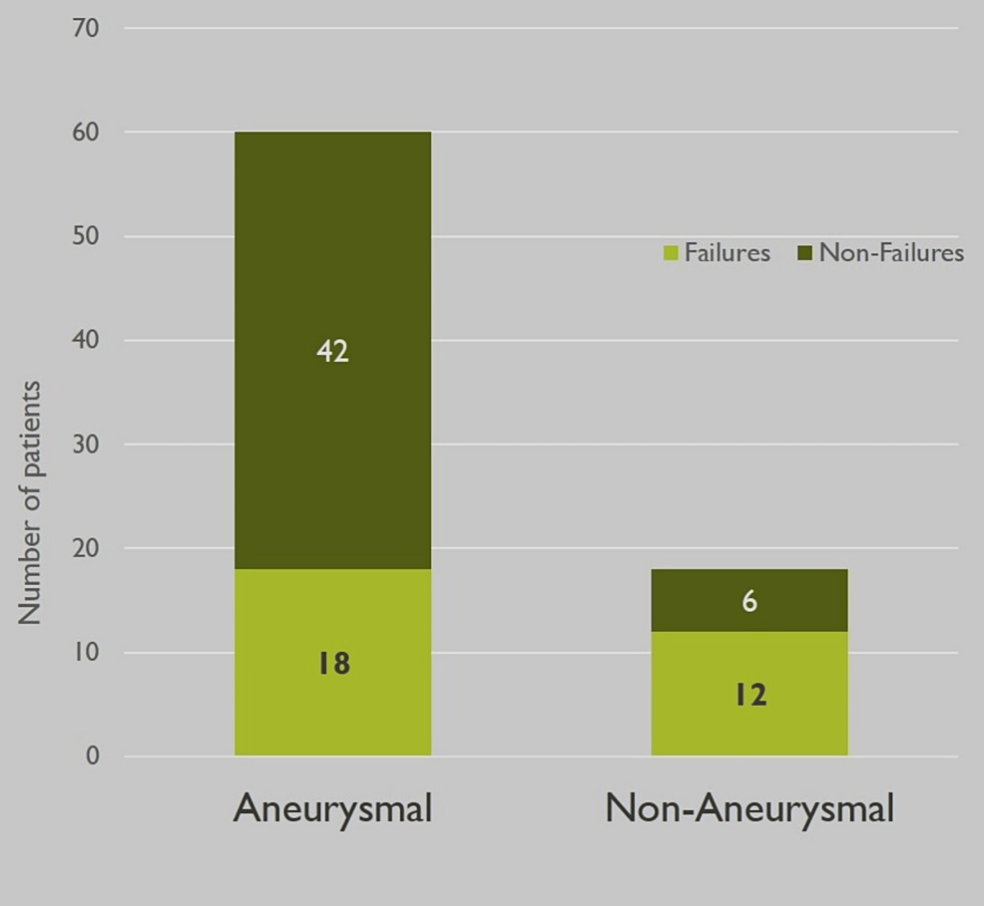
Rates of EVD failures in aSAH and naSAH patients This figure presents the amount of external ventricular drain (EVD) failures in both aneurysmal subarachnoid hemorrhage (aSAH) and non-aneurysmal subarachnoid hemorrhage (naSAH) patients (p < 0.001).

Among SAH patients with EVD placement, 18 aSAH, and 12 naSAH had EVD wean failures. 11 aSAH and five naSAH patients required ventriculoperitoneal shunt (VPS) placement. The incidence of VPS placement among all EVD patients in our study was 20.5%. A chi-square analysis was performed, and aSAH patients were found to have higher rates of VPS/ETV placement (p<0.001) and EVD wean failures (p<0.001) than naSAH patients. Values were considered if p < 0.05. These results are represented in Figures 4, 5. In addition, a one-sided z-test was run to compare the proportion of patients undergoing VPS at our institution to patients undergoing VPS in the study by Lewis and Kimberly, with a p-value of 0.07 [11].

## Discussion

Standardized guidelines to begin EVD weaning are limited [7]. Our study was performed to analyze the protocol utilized at our institution and to share our criteria for initiating the EVD weaning process. Our institution follows a gradual weaning process that was developed over the course of 8 years and has not been modified since the start of this retrospective study. The purpose of these criteria is to provide an approach to beginning the EVD weaning process that is simple to follow while optimizing outcomes. These criteria allow medical students, resident/fellow physicians, nursing staff, physician assistants, and physicians to make informed decisions for the management of SAH requiring EVD. Our protocol provides a broad framework of EVD management that can be particularly useful in smaller non-academic centers as well as developing countries with limited resources and scarce specialist availability. These criteria reduced infection rates, length of stay (LOS), and VPS/ETV placement in our patient population.

In Dasenbrock, et al., an extensive nationwide analysis of hospital-acquired infections in patients with aSAH was performed, however, infection rates in EVD patients alone were not analyzed [12]. Their study population included patients with and without EVDs. UTIs, respiratory infections, and meningitis rates were found to be 24%, 23%, and 4.4%, respectively. When comparing our aSAH patient population with and without EVD, rates of urinary tract infections (UTIs), respiratory infections, and meningitis were 26%, 31%, and 3% respectively. Several reasons for an elevated UTI rate compared to the UTI rate found in Dasenbrock, et al. include our significantly smaller sample size (total of 100 aSAH compared to 7516 aSAH patients) as well as differences in mean age and percentage of female patients. In our analysis, an association between gender and UTIs was also found (88% of female patients had UTIs, and 100% of *Escherichia coli* urinary tract infections were in female patients). A potential confounding variable for UTIs such as diabetes in a rural, southern United States population which our institution treats was not accounted for. Other potential confounding variables such as age and sexual activity were also not accounted for. Similarly, the higher pneumonia rates in our population could be due to the smaller sample size, severity of the SAH in certain patients requiring intubation, and underlying comorbidities in our patient population such as congestive heart failure or chronic obstructive pulmonary disease. In our study, we had a lower rate of meningitis compared to the national average, and this could be due to differences in SAH severity as well as non-infectious complications. Another factor that could influence infection rates is length of stay. Although limited data exists on the average length of stay for patients with SAH and naSAH requiring EVDs, our institution’s length of stay for aSAH with EVDs is 20.35 compared to 22 days according to a recently published study from another academic institution [13].

Lewis and Taylor Kimberly conducted a study at Massachusetts General Hospital which found that ~30% (35/116) of SAH patients requiring an EVD also required ventriculoperitoneal shunt (VPS) placement [11]. Their sample size was 116 patients whereas our sample size was 78 patients. Only 21% (16 of 78 of SAH patients) required VPS placement following our EVD wean initiation criteria. At both institutions, an EVD wean failure was defined as lowering or opening the EVD due to elevated ICP, decline in clinical status, radiologic finding of hydrocephalus, or leakage from the EVD site. Although significance was not found with a p-value of 0.07, this was an important finding. A simple, uniform approach to deciding when to begin EVD weaning may result in lower VPS placement rates in EVD patients across the nation. 

Table 2 displays the initiation criteria that were used. The first criterion ensures that the underlying cause that produced the placement of an EVD has resolved which includes securing the aneurysm if present. The second criterion ensures that bleeding is no longer persistent. The third criterion ensures that hydrostatic pressure has decreased after regeneration of arachnoid villi on the epithelial layer of ventricles allowing for natural ICP regulation via CSF absorption. The fourth criterion provides a range of ICPs that is within homeostatic limits reinforcing the third criterion that the patient has begun to recover and will be able to reabsorb CSF and regulate ICP naturally [14]. The fifth criterion ensures that the patient is neurologically stable with a reduced chance of redeveloping neurological symptoms, once again ensuring patient safety. These criteria are a mix of objective clinical and radiographic findings that can be broadly applied to patients with different underlying comorbidities, different demographic makeup, and at different institutions with varying levels of resources.

**Table 2:** External ventricular drain (EVD) weaning criteria CSF: cerebrospinal fluid; ICP: intracranial pressure Acceptable to wean when a patient has mild cerebral vasospasm but not moderate to severe cerebral vasospasm.

EVD Weaning Initiation Criteria
Resolution of the reason for placement.
CSF output is clear (non-bloody).
CSF output is less than 250 ml over 24 hours.
ICP within normal ranges of 5 to 20.
Stable Neurological Exam.

A limitation of this study includes population risk factors. Our institution is a tertiary medical center for much of rural Georgia, home to many people who suffer from risk factors including diabetes, hypertension, and smoking [15]. Another limitation of this study is the patient sample size. Despite analyzing three years of the SAH patient population at our institution, only 151 patients were included in this retrospective study and there were unequal arms amongst aSAH and naSAH patients.

Another point of discussion that will be at the forefront of EVD weaning is the incorporation of a rapid EVD wean versus a traditional gradual EVD wean. Some institutions have begun using a rapid weaning protocol which reduces hospital length of stay resulting in lowering both cost and hospital-acquired infections [11]. However, as a recent study showed, there are varying protocols on what that weaning protocol entails, with many institutions immediately clamping the drain regardless of height, while other institutions allow 48 hours after an attempted wean trial before clamping. As more institutions adopt rapid weaning protocols and there is further evidence pointing to which type of rapid wean strategy might be most effective, we would also like to further investigate how our weaning initiation protocol can be adapted to a rapid EVD wean strategy. Although our study analyzed EVD weaning using the gradual EVD wean approach, our principles have the potential to be applied when initiating a rapid wean. Our next study focus will be on investigating these same 5 criteria to begin an EVD wean, with subsequent initiation of a rapid EVD wean with a 5 mmHg increase in height every 12 hours (as opposed to 24 hours). Our goal in sharing these criteria to initiate the weaning process is to not only facilitate safe and efficient EVD weaning but also to be a stepping stone for further studies and analyses of different EVD weaning strategies in subarachnoid hemorrhage patients, including the rapid wean approach.

## Conclusions

The criteria used at our institution provided comparable hospital-acquired infection rates and intensive care unit length of stay with a lower ventriculoperitoneal shunt placement compared to national averages. These recommendations provide simple and effective standardized criteria to begin the EVD removal process and may be used by medical students, residents, fellows, advanced practice providers, and attending physicians to utilize. The findings of this study suggest that these criteria should be further investigated in larger, randomized controlled trials. 

## References

[1] Sweeney K, Silver N, Javadpour M (2016). Subarachnoid haemorrhage (spontaneous aneurysmal). BMJ Clin Evid.

[2] Capion T, Lilja-Cyron A, Juhler M, Mathiesen TI, Wetterslev J (2020). Prompt closure versus gradual weaning of external ventricular drainage for hydrocephalus in adult patients with aneurysmal subarachnoid haemorrhage: a systematic review. BMJ Open.

[3] Palasz J, D'Antona L, Farrell S, Elborady MA, Watkins LD, Toma AK (2022). External ventricular drain management in subarachnoid haemorrhage: a systematic review and meta-analysis. Neurosurg Rev.

[4] Akinduro OO, Vivas-Buitrago TG, Haranhalli N (2020). Predictors of ventriculoperitoneal shunting following subarachnoid hemorrhage treated with external ventricular drainage. Neurocrit Care.

[5] Vale FL, Bradley EL, Fisher WS 3rd (1997). The relationship of subarachnoid hemorrhage and the need for postoperative shunting. J Neurosurg.

[6] Fried HI, Nathan BR, Rowe AS (2016). The insertion and management of external ventricular drains: an evidence-based consensus statement : a statement for healthcare professionals from the Neurocritical Care Society. Neurocrit Care.

[7] Gigante P, Hwang BY, Appelboom G, Kellner CP, Kellner MA, Connolly ES (2010). External ventricular drainage following aneurysmal subarachnoid haemorrhage. Br J Neurosurg.

[8] Modi S, Shah K, Schultz L, Tahir R, Affan M, Varelas P (2019). Cost of hospitalization for aneurysmal subarachnoid hemorrhage in the United States. Clin Neurol Neurosurg.

[9] Abulhasan YB, Alabdulraheem N, Schiller I, Rachel SP, Dendukuri N, Angle MR, Frenette C (2018). Health care-associated infections after subarachnoid hemorrhage. World Neurosurg.

[10] Chung DY, Thompson BB, Kumar MA (2022). Association of external ventricular drain wean strategy with shunt placement and length of stay in subarachnoid hemorrhage: a prospective multicenter study. Neurocrit Care.

[11] Lewis A, Taylor Kimberly W (2014). Prediction of ventriculoperitoneal shunt placement based on type of failure during external ventricular drain wean. Clin Neurol Neurosurg.

[12] Dasenbrock HH, Rudy RF, Smith TR (2016). Hospital-acquired infections after aneurysmal subarachnoid hemorrhage: a nationwide analysis. World Neurosurg.

[13] Nelson SE, Suarez JI, Sigmon A, Hua J, Weiner C, Sair HI, Stevens RD (2022). External ventricular drain use is associated with functional outcome in aneurysmal subarachnoid hemorrhage. Neurol Res Pract.

[14] Lun MP, Monuki ES, Lehtinen MK (2015). Development and functions of the choroid plexus-cerebrospinal fluid system. Nat Rev Neurosci.

[15] Russell S, Sturua L, Li C (2019). The burden of non-communicable diseases and their related risk factors in the country of Georgia, 2015. BMC Public Health.

